# Novel Insights into the Genetic Diversity of *Balantidium* and *Balantidium*-like Cyst-forming Ciliates

**DOI:** 10.1371/journal.pntd.0002140

**Published:** 2013-03-28

**Authors:** Kateřina Pomajbíková, Miroslav Oborník, Aleš Horák, Klára J. Petrželková, J. Norman Grim, Bruno Levecke, Angelique Todd, Martin Mulama, John Kiyang, David Modrý

**Affiliations:** 1 Department of Pathology and Parasitology, Faculty of Veterinary Medicine, University of Veterinary and Pharmaceutical Sciences, Brno, Czech Republic; 2 Biology Centre of the Academy of Sciences of the Czech Republic, Institute of Parasitology, České Budějovice, Czech Republic; 3 University of South Bohemia, Faculty of Science, České Budějovice, Czech Republic; 4 Institute of Vertebrate Biology, Academy of Sciences of the Czech Republic, Brno, Czech Republic; 5 Liberec Zoo, Liberec, Czech Republic; 6 Department of Biological Sciences, Northern Arizona University, Flagstaff, Arizona, United States of America; 7 Laboratory for Parasitology, Faculty of Veterinary Medicine, Ghent University, Ghent, Belgium; 8 Centre for Research and Conservation, Royal Zoological Society of Antwerp, Antwerp, Belgium; 9 World Wildlife Fund, Bangui, Central African Republic; 10 Sweetwaters Chimpanzee Sanctuary, Ol Pejeta Conservamcy, Nanyuki, Kenya; 11 Limbe Wildlife Centre, Limbe, Cameroon; 12 CEITEC – Central European Institute of Technology, University of Veterinary and Pharmaceutical Sciences, Brno, Czech Republic; Georgetown University, United States of America

## Abstract

Balantidiasis is considered a neglected zoonotic disease with pigs serving as reservoir hosts. However, *Balantidium coli* has been recorded in many other mammalian species, including primates. Here, we evaluated the genetic diversity of *B. coli* in non-human primates using two gene markers (SSrDNA and ITS1-5.8SDNA-ITS2). We analyzed 49 isolates of ciliates from fecal samples originating from 11 species of captive and wild primates, domestic pigs and wild boar. The phylogenetic trees were computed using Bayesian inference and Maximum likelihood. *Balantidium entozoon* from edible frog and *Buxtonella sulcata* from cattle were included in the analyses as the closest relatives of *B. coli*, as well as reference sequences of vestibuliferids. The SSrDNA tree showed the same phylogenetic diversification of *B. coli* at genus level as the tree constructed based on the ITS region. Based on the polymorphism of SSrDNA sequences, the type species of the genus, namely *B. entozoon*, appeared to be phylogenetically distinct from *B. coli*. Thus, we propose a new genus *Neobalantidium* for the homeothermic clade. Moreover, several isolates from both captive and wild primates (excluding great apes) clustered with *B. sulcata* with high support, suggesting the existence of a new species within this genus. The cysts of *Buxtonella* and *Neobalantidium* are morphologically indistinguishable and the presence of *Buxtonella*-like ciliates in primates opens the question about possible occurrence of these pathogens in humans.

## Author Summary

*Balantidium coli* is a pathogenic ciliate occurring in various hosts, including primates. Balantidiasis is considered a neglected disease with zoonotic potential and it is associated with pigs as reservoirs. Although it is considered to be rare, a high prevalence of *B. coli* persists in tropical and subtropical areas. The infection can cause symptoms ranging from mild diarrhea to fulminating dysentery. Recently, balantidiasis has appeared to be a serious problem in immunocompromised persons. Although a chapter about balantidiasis is included in almost every parasitology textbook, many aspects of the occurrence of these ciliates in mammals, including humans, remain unknown. As balantidiasis can pose a serious health problem, a better understanding of this organism has important practical implications. We performed an extensive evaluation of genetic diversity in *B. coli* based on samples from non-human primates, together with comparative material from pigs and reference sequences. We find that the diversity of the cyst-forming ciliates in non-human primates is broader than expected. This discovery poses novel questions about the real spectrum of intestinal ciliates occurring in humans, and might explain unknown factors in the epidemiology and pathogenicity of ciliate infections.

## Introduction

*Balantidium coli* (Vestibuliferida: Balantidiidae) is a cosmopolitan ciliate colonizing the intestine of many mammalian hosts. However, domestic pigs and wild boars are considered to be the principal host and major reservoir [1], [2]. Balantidiasis is considered a zoonotic disease and human clinical cases in developed countries were typically associated with close contact with pigs in the past [2], [3]. Nowadays, localities with a high prevalence of *B. coli* infection in humans persist mostly in tropical and subtropical areas [4], [5]. Apart from humans, *B. coli* is also commonly reported to infect both captive and free-living non-human primates [6], [7], [8].

The clinical importance of *B. coli* varies. Presently, human populations living in close proximity with domestic pigs are naturally resistant and mostly without any clinical manifestation [5]. However, the infection can cause disease, with symptoms ranging from mild diarrhea to fulminating dysentery. On rare occasions these organisms may also invade other organs [2], [9], [10], [11], which is more frequently observed in immunocompromised individuals afflicted with AIDS or leukemia [12], [13].

The *Balantidium* taxonomy is somewhat controversial due to the pleomorphism of its trophozoites [2], [14], [15] and range of its hosts. *Balantidium coli* observed in dysenteric patients was originally described as *Paramecium coli* by Malmstein (1857) [16]. Subsequently, Stein (1863) [17] reclassified the ciliate into the genus *Balantidium*, which was erected a few years earlier by Claparède and Lachmann (1858) [18] for the newly described *Balantidium entozoon* from frogs. A great majority of the taxa included in this genus were isolated from amphibian, fish or insect hosts [19]. In mammals, all balantidia are currently referred to as *B. coli*, despite that several other species of *Balantidium* have recently been described based on slight morphological differences in trophozoites. The broad synonymy of *B. coli* includes twelve other species of mammalian balantidium, specifically from primates, pigs, guinea pigs and camels [14], [20], [21]. Apparently, trophozoite morphology alone is insufficient for taxonomical purposes.

The worldwide distribution of *B. coli* in various hosts, together with the zoonotic potential and unclear epidemiology of human balantidiasis, calls for further study addressing the genetic diversity of these pathogens. Currently, fairly extensive and congruent molecular phylogenies have been obtained based on small ribosomal subunits of free-living and commensal ciliates in recent years [22], [23], [24]. The SSrRNA is a gene of taxonomic relevance at the genus level and, nowadays, it is broadly used for taxonomic studies in combination with morphological features [e.g. 25]. Currently, only few SSrRNA sequences of *Balantidium* are available in the GenBank and their comparison across various hosts, pigs, ostriches and gorillas, has revealed little variability [26]; however, the sequence of *B. entozoon* differs by 5% from those of *B. coli* [27].

Recently, the molecular diversity of *B. coli* at the species/subspecies level has been explored based on the hypervariable gene marker ITS1-5.8S rRNA-ITS2 [21], leading to designation of two different genotypes, A and B, in isolates from domestic pigs and ostriches. However, a later study by the same authors demonstrated that the “genotypes” are *de facto* genetic variants, representing at least two separate micronuclear rRNA genes, which can be present even within a single cell of *B. coli* [28]. Moreover, they distinguished five types of these main variants (A0, A1, A2, B0 and B1) on the basis of the ITS1 helix II characteristics. Such ambiguous polymorphism questions the applicability of internal transcribed spacers as markers for analyzing the intraspecific genetic variability of *B. coli* and any taxonomical or epidemiological implications.

Our previous research on the occurrence of *B. coli* in African great apes revealed the common presence of trophozoites and/or cysts in captive animals from European facilities and African sanctuaries, in contrast to its absence in wild populations [7]. Although balantidiasis is a neglected disease, molecular diversity of *Balantidium* in humans has not yet been addressed. Based on the samples from captive and wild non-human primates, together with comparative material from domestic pigs in Europe and tropical Africa, we performed an extensive evaluation of the genetic diversity of *B. coli* using two nuclear gene markers: SSrDNA and ITS1-5.8SrDNA-ITS2. Our phylogenetic analyses also comprise sequences from type species of the genus ( = *B. entozoon*), and for the first time also the closely related ciliate from cattle, *Buxtonella sulcata*, to address the potential polyphyletic character of the genus *Balantidium*.

## Materials and Methods

### Fecal samples

Our study was completely non-invasive including only examination of fecal samples. The only invasive sampling was the use of cloacal lavage in edible frogs. This material was collected prior to our research during the field course for undergraduate students. As such, it was approved by Administratia Rezervatiei Biosferei Delta Dunarii (for details about the permits and collaborating authorities see Text S1).

Fecal samples from captive primates were obtained in collaboration with (i) several European Zoos and (ii) African sanctuaries during routine diagnostic check-ups. Every facility was provided with the results, which they later used for antiparasitic control. In European countries, our research was approved by particular Zoos (which follow their respective national animal care regulations or guidelines). To our knowledge, and based on long-term research on primate parasites, such non-invasive fecal sampling has no other regulations in the EU. Thus, this study complies with individual national animal care regulations or guidelines.

Fecal samples of primates (n = 35) containing *B. coli*-like ciliates were selected based on coproscopic analysis performed in previous pilot studies [7], [29]; these samples and their origin are listed in Tables 1 and 2. Other material included fecal samples from domestic pigs containing *B. coli*, cattle containing *Buxtonella sulcata* (mostly cysts and few trophozoites based on coproscopic analysis) and edible frogs positive for *Balantidium entozoon* (the samples and their origin are listed in Table 3). In captive facilities, fecal samples of primates, pigs and cattle were collected individually and by animal keepers during routine daily cleaning of cages/sleeping quarters. In the wild, fecal samples were obtained during distance tracking of the animals. Fecal samples of edible frogs were obtained by cloacal lavage. All samples were analyzed coproscopically using Sheather's flotation and Merthiolate-iodine-formaldehyde (MIFC) sedimentation [7].

**Table 1:** The summary of isolates from captive African great apes.

Site (State)	Host species	Origin	S. No.	A. No. 18S	A. No. ITS
Antwerp Zoo (Belgium)	chimpanzees (*Pan troglodytes*)	captivity	1	JQ073313	JQ073351
Dierenpark Amersfoort (Netherlands)	chimpanzees (*Pan troglodytes*)	captivity	1	JQ073311	JQ073349
La Vallée des Signes (France)	chimpanzees (*Pan troglodytes*)	captivity	1	JQ073309	JQ073347
Limbe Wildlife Centre (Cameroon)	chimpanzees (*Pan troglodytes*)	captivity	3	JQ073315-17	JQ073353-55
Ogród Zoologiczny w Opolu (Poland)	gorilla (*Gorilla gorilla*)	captivity	1	JQ073312	JQ073350
Sweetwaters Chimpanzee Sanctuary (Kenya)	chimpanzees (*Pan troglodytes*)	captivity	3	JQ073314, -31-32	JQ073352, -76-77
Chimps' Sanctuary, PN de Conkouati Douli (Republic of Congo)	chimpanzees (*Pan troglodytes*)	captivity	3	JQ073329	JQ073372-74
Twycross Zoo (GB)	chimpanzees (*Pan troglodytes*)	captivity	2	JQ073310	JQ073348, -18
Zoo Aquarium Madrid (Spain)	chimpanzees (*Pan troglodytes*)	captivity	2	JQ073307-08	JQ073345-46
Zoologischer Garten Leipzig (Germany)	bonobo (*Pan paniscus*)	captivity	1	JQ073306	JQ073344

NR- National Reservation; PN-Park National; S. No – number of samples; A. No. 18S/ITS – accession numbers for 18S-rDNA/ITS1-5.8SrRNA-ITS2 sequences in the GenBank.

**Table 2:** The summary of isolates from captive and wild-ranging other primates.

Site (State)	Host species	Origin	S. No.	A. No. SSU	A. No. ITS
Amsterdam Zoo (Nl)	mandrill (*Mandrillus sphinx*)	captivity	1	-	JQ073338
	celebes crested macaque (*Macaca nigra*)	captivity	1	-	JQ073382
AAP Sanctuary For Exotic Animals (Nl)	hamadrys baboon (*Papio hamadryas*)	captivity	6	JQ07333	JQ073339-41,-75,-81,-84
	barbary macaque (*Macaca sylvanus*)	captivity	1	-	JQ073369
Safari Park Beekse Bergen (Nl)	rhesus macaque (*Macaca mulatta*)	captivity	2	JQ073327-28	JQ073367-68
Apenheul Primate Park (Nl)	lion-tailed macaque (*Macaca silenus*)	captivity	3	-	JQ073370-71,-83
Sweetwaters Chimpanzee Sanctuary (K)	hamadrys baboon (*Papio hamadryas*)	captivity	1	JQ073305	JQ73343
Dzanga-Ndoki NP (CAR)	agile mangabey (*Cercopithecus agilis*)	wild	2	JQ073325,-36	JQ073364,-85

Nl-Netherlands, K-Kenya, CAR-Central African Republic; S. No. – number of samples; A. No. 18S/ITS – accession numbers for 18S-rDNA/ITS1-5.8SrRNA-ITS2 sequences in the GenBank.

**Table 3:** The summary of isolates from domestic pigs, cattle and edible frogs.

Site (State)	Host species	S. No.	A. No. 18S	A. No. ITS
Czech Republic	domestic pig (*Sus scrofa domestica*)	5	JQ073304,-21-24	JQ073342,-60-63
Madagascar	domestic pig (*Sus scrofa domestica*)	3	JQ073320	JQ073357-59
Cameroon	domestic pig (*Sus scrofa domestica*)	2	JQ073334	JQ073379-80
Kenya	domestic pig (*Sus scrofa domestica*)	1	JQ073333	JQ073378
Central African Rep.	domestic pig (*Sus scrofa domestica*)	2	JQ073326	JQ073365-66
Czech Republic	wild boar (*Sus scrofa*)	1	JQ073319	JQ073356
Belgium	cattle (*Bos taurus*)	2	JQ073337	JQ073386-87
Romania	edible frog (*Pelophylax* kl. *esculentus*)	2	JQ408692	JQ408693-94

S. No.-number of samples; A. No. 18S/ITS – accession numbers for 18S-rDNA/ITS1-5.8SrRNA-ITS2 sequences in the GenBank.

### DNA isolation, PCR, sequencing

Fecal samples were fixed in 96% ethanol. A portion of each sample was dried overnight at 37°C. Total DNA was extracted using the Invitrogen Stool Kit following the manufacturer's instructions. Primers for amplification of the SSrRNA gene of *B. coli*, *B. sulcata* and *B. entozoon* were designed based on the published sequences of SSrDNA in the GenBank and also of other ciliates that could be present in fecal samples. The partial sequence of SSrDNA with the sizes 1047 bp (*B. coli, B. sulcata*) and 1040 bp (*B. entozoon*) were amplified using the forward primer SSUf (5′-CGCAAATCGCGATTTTGTCGCG-3′) and either the reverse primer SSUrBB (5′-AAATACATAGTCCCTCTAAGAAGTC-3′) for *B. coli* and *B. sulcata* or SSUrBE (5′-CCCTCTAAGAAGCTAATACTC-3′) for *B. entozoon*. PCR was performed in a Biometra T-personal thermocycler (Schoeller) programmed as follows: (i) SSUf+SSUrBB: 10 min at 94°C, 35 cycles of 1 min at 94°C, 1 min at 59°C, 1 min at 72°C, and 5 min at 72°C, and (ii) SSUf+SSUrBE: 10 min at 94°C, 35 cycles of 1 min at 94°C, 1 min at 53°C, 1 min at 72°C, and 5 min at 72°C. The ITS1 - 5.8S-rRNA - ITS2 coding region was amplified using published primers B5D and B5R for all three species and the PCR program according to the protocol [21]. The PCR products were checked by electrophoresis using 12 µl of PCR reaction loaded into a 1% agarose gel run using the Vilber Lourmat electrophoresis system (Schoeller); PCR products were visualized by staining with Goldview and UV translumination. The amplicons were purified using the Qiagen extraction gel kit. Sequencing of these amplicons was performed by Macrogen, Korea. Each sample was sequenced in both directions and the sequence contigs were assembled using the program ChromasPro, version 1.5 (Technelysium Pty Ltd). BLAST analyses were performed for all new sequences.

### Phylogenetic analyses

**Figure 1:**
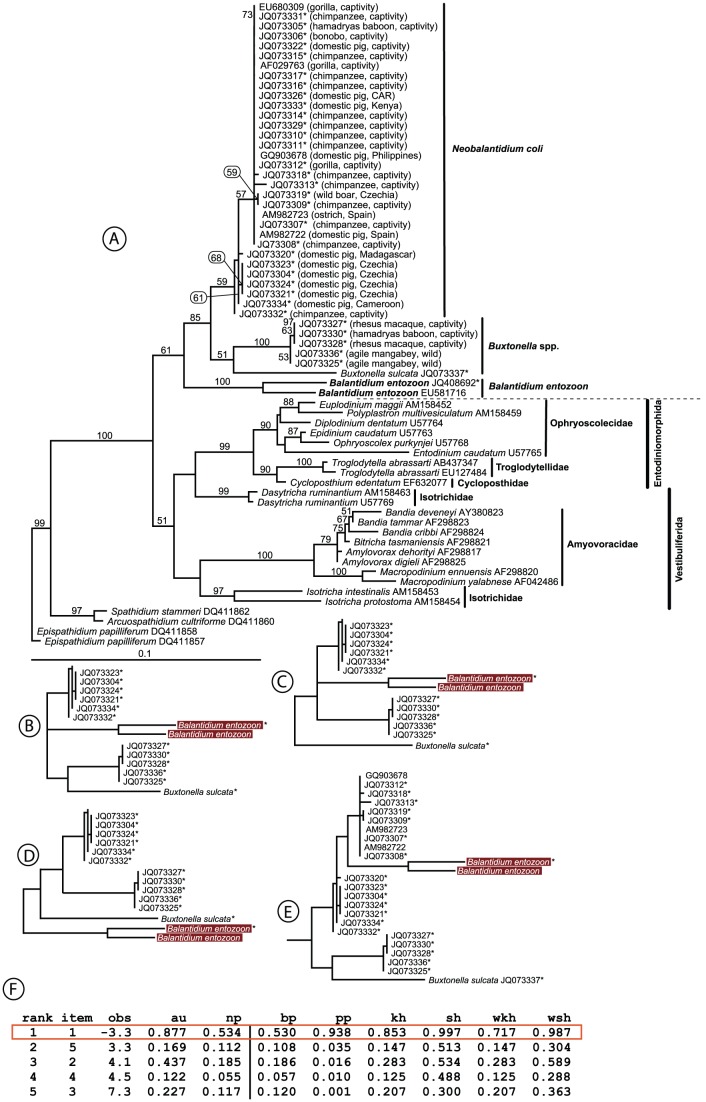
SSrDNA tree based on Trichostomatia SSrDNA sequences computed by RAxML. A. The numbers above the branches represent Maximum likelihood bootstrap supports as computed from 1000 replicates. The scale bar represents 10 changes per 100 positions. B–E. The tree is complemented by an AU topology test, with all tested topologies shown below the main tree (topologies shown in reduced form). New sequences are marked with a star.

rRNA gene loci sequences coding for both SSU and ITS were aligned with available homologues from GenBank using the Kalign program at http://www.ebi.ac.uk/Tools/msa/kalign/ [30], [31]. Both alignments were manually edited in BioEdit [32] and ambiguously aligned. In the case of ITS sequences, the ambiguously aligned regions were omitted from further analyses. Alignments were analyzed individually and also in a combined data set. SSU and ITS data sets were analyzed using both Maximum likelihood (ML) and Bayesian inference (BI). Maximum likelihood topologies were computed using a gamma corrected GTR model of evolution and SPR heuristics as implemented in PhyML 3.0 [33]. Branching support was estimated from 1000 non-parametric bootstrap replicates using the above mentioned software and conditions. Bayesian topologies were estimated using Phylobayes 3.2 [34] under the GTR and/or GTR-CAT model of evolution. The latter is a combination of the empirical mixture model of 40 profiles (C40) sharing exchange rates as defined by the GTR model. For all data sets, two independent chains were run until they converged (i.e. their maximum observed discrepancy was lower than 0.1) and the effective sample size of all model characteristics was at least 100. Bayesian posterior probabilities (PP) provide branching support for the resulting topology (only branches with a PP higher than 0.95 were considered to be supported by the models). The possible hypotheses for the evolutionary history of the genus *Balantidium* were tested using the approximately unbiased (AU) test [35] on the SSrRNA gene dataset. Alternative topologies of the in-group (Fig. 1) were constrained and re-optimized using RAxML [36]. The site-specific log-likelihood scores of the resulting trees were obtained by the same software and further analyzed using the AU-test as implemented in CONSEL [37].

## Results

The sequences of both above-mentioned markers obtained in this study from each host are available in GenBank under the accession numbers JQ73304-87, JQ408692-94 (Table 1–3).

### Molecular phylogenies of SSrRNA genes/primary structure

Thirty-five partial SSrDNA sequences of ciliate species were examined and compared to other SSrDNA sequences representing the genus *Balantidium* (EU680309, AM982722-23, AF029763, GQ903678, EU581716) and related ciliates classified within the subclass Trichostomatia (AB437347, AF298817, AF298820-21, AF298823-25, AF042486, AM158452-54, AM158459, AM158463, AY380823, EF632077, EU127484, U57763-65, U57768-69) and Haptoria (DQ411862, DQ411860). This tree was rooted using *Epispathidium papilliferum* (DQ411857-8) as an outgroup.

The maximum likelihood (RAxML with GTR model) topology of our resulting tree shows that the subclass Trichostomatia is a monophyletic and highly supported group divided into two major clades. However, the order Vestibuliferida appears to be paraphyletic in its origin (Fig. 1). **Clade I** includes all the sequences of *Balantidium* and is further divided into two sub-clades: one that includes sequences of *Balantidium coli, Buxtonella sulcata* and *Buxtonella*-like ciliates from mammals, and the other represented by *B. entozoon* isolates only. Although the topology inferred from SSrRNA genes is unstable, it is very likely that the genus *Balantidium* is polyphyletic. Such topology is strongly preferred by the AU test (Fig. 1), although none of the alternative topologies could be fully rejected (see Fig. 1 for details). Generally, *B. entozoon* isolates jump to different positions in the tree, based on the particular method and substitutional model used. However, all obtained SSrDNA based topologies (RAxML with GTR model; PhyloBayes with CAT model; PhyloBayes with GTR model, see dataset S1, S2, S3 for details) result in the polyphyly of the genus *Balantidium*. All our SSrDNA sequences from pigs, wild boar, great apes and a single sequence from a captive baboon (JQ073305), together with reference sequences of *Balantidium*, were grouped into a single clade corresponding to *B. coli*. This clade displays internal polytomy, obviously due to the presence of a high number of similar sequences. Several ciliates from primates (agile mangabeys: JQ073325 and JQ073336; rhesus macaques: JQ073327-28; the hamadryas baboon: JQ0733330) formed a separate clade that branched together with sequences of *B. sulcata* from cattle. However, such a relationship is only very weakly supported by ML trees; trees computed by PhyloBayes placed *B. sulcata* on a long branch inside the *B. coli* clade (see dataset S1, S2).

All other trichostomatids are included in the main second clade—**clade II**—that is separated into two sub-clades with a weak bootstrap value (51%). One sub-clade contains mainly Australian intestinal ciliates and some isotrichids (Vestibuliferida) and with high support values of both branches (100%, 97%). The second sub-clade is represented by Ophryoscolecidae, Troglodytellidae and Cycloposthidae (Entodiniomorphida), plus one isotrichid ciliate (Vestibuliferida), with a support value of 99% (Fig. 1). Both ML and Bayesian inference showed similar clade II topologies, albeit with numerous polytomies in the Bayesian tree (see Fig. 1 and datasets S1, S2, S3 for details).

### Molecular phylogenies of ITS1-5.8S rRNA-ITS2 marker

**Figure 2:**
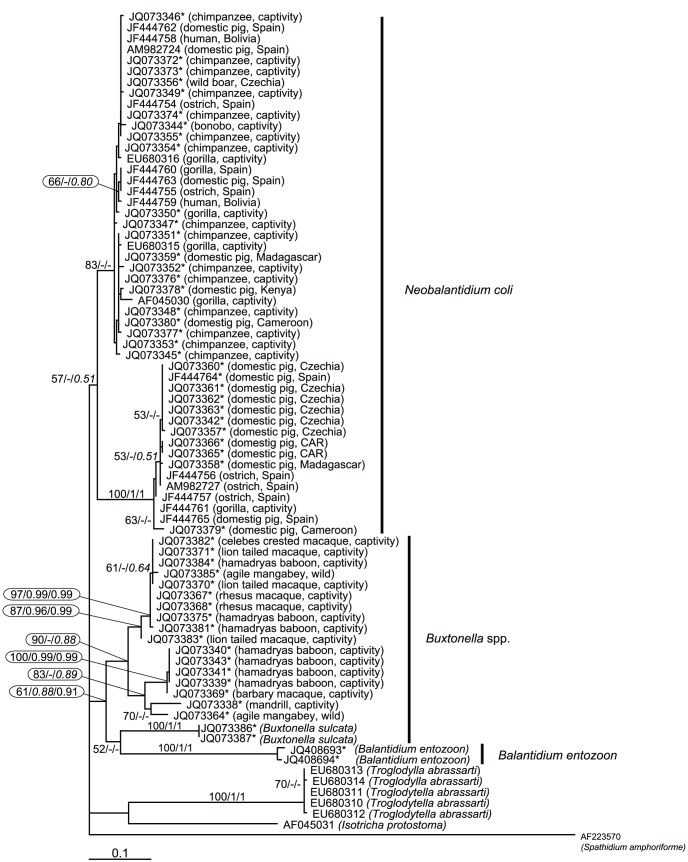
Maximum likelihood phylogenetic tree as inferred from the ITS1-5.8S-ITS2 DNA region. The tree was computed using PhyML with the GTR model for nucleotide substitutions. Numbers above branches indicate ML bootstrap support from 1000 replicates/PhyloBayes posterior probabilities computed with CAT model/PhyloBayes posterior probabilities computed with GTR model. New sequences are marked with a star.

The phylogenetic trees based on the ITS1-5.8SrRNA-ITS2 sequences were estimated using Maximum likelihood phylogeny (PhyML) with the GTR model and Bayesian inference (PhyloBayes) with CAT and GTR models. This sequence data set includes our 52 sequences and reference sequences from GenBank that are comprised of five isolates of *T. abrassarti* (EU680310-14), and one from *I. prostoma* (AF045031); the haptorian ciliate *Spathidium amphoriforme* (AF223570) was used as an out-group. The resulting ML tree topology shows three major groups: (i) with *T. abrassarti* and *I. prostoma*, (ii) including *B. entozoon*, *B. sulcata* and isolates of ciliates from other primates and (iii) the “typical” *B. coli* clade (Fig. 2).

The majority of the supposed *B. coli* isolates were placed into a single clade, which was further divided into two highly supported sub-clades corresponding to the two major A and B genetic variants of *B. coli* as proposed [28]. The A-group includes 21 of our ciliate sequences and 11 reference sequences from captive great apes, two sequences from humans, one from wild boar, two from ostriches and six sequences from domestic pigs. The B-group contains 10 newly obtained sequences from ciliates from domestic pigs from different topodemes and four reference sequences (3 isolates from ostriches: AM982727, JF444756-7; captive gorilla: JF444761 and 2 domestic pigs). All obtained sequences originating from domestic pigs belong to type of genetic variant B. The sequence JQ073379 from the Cameroonian domestic pig branched separately in genetic variant B, which is most probably a new type of genetic variant. In three out of five populations of domestic pigs, both main types detected (Cameroon, Czech Republic, Madagascar), while in Kenya and CAR only one of them was found (either A or B, Fig. 2).

Importantly, the sequences of all 17 isolates of ciliates obtained from primates other than great apes and humans, together with *B. sulcata* and *B. entozoon*, formed a clade separated from *B. coli*. This group of isolates from primates is further divided into two strongly supported sub-groups that may represent a type of genetic variants. In this analysis, *B. entozoon* clustered as a sister taxon to *B. sulcata*, although with poor support. Bayesian trees inferred from ITS (CAT and GTR models) showed the same topology with the main clades forming polytomies in the tree. However, the ITS-based ML tree topology corresponds well to that of the SSrDNA-based tree, which was preferred by the AU test.

Eight chromatograms of the ITS1-5.8S rRNA-ITS2 sequences of *B. coli* revealed multiple sequence signals, specifically three chimpanzees (JQ 073346, JQ073348, JQ073354), three domestic pigs (JQ073357, JQ073378, JQ073380), a single bonobo (JQ073344) and a wild boar (JQ073356), suggesting the presence of more genetic variants within one isolate. The same situation is also seen in primates other than great apes, where five sequences with multiple signals were found. This situation, in agreement with results of a previous study [28], could not be resolved without cloning and is further discussed below.

### Taxonomic summary

Our analyses have proven the polyphyletic character of the current genus *Balantidium* Claparède and Lachmann, 1858. The genus is well typified by its type species *Balantidium entozoon* from ranid frogs *Pelophylax* kl. *esculentus*. However, the historical placement of *Paramecium coli* Malmstein, 1857 into this genus by Stein (1863) is not supported by our results. As there is no available alternative in the complex synonymy of *Balantidium coli*, we erect a new genus to accommodate *Paramecium coli* Malmstein, 1857, together with other species previously described as members of the genus *Balantidium* from homeothermic hosts.

### *Neobalantidium* gen. nov

**Type species:** *Paramecium coli* Malmstein, 1857.

**Etymology:** the new generic name is created by the prefix “neo-“, meaning new/novel. As there are no pathological signs associated with the presence of balantidia in amphibians, we suggest continuing to use the term “balantidiasis” for describing the clinical disease caused by species of *Neobalantidium* in homeothermic vertebrates including humans.

**Figure 3:**
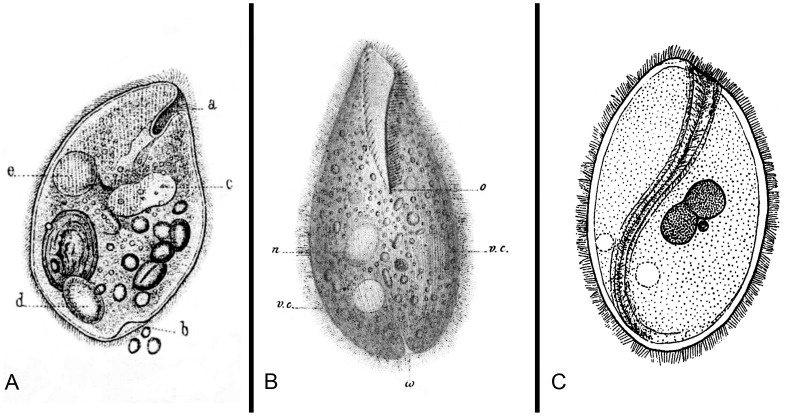
Reproduction of the original drawings of trophozoites of *Paramecium coli*, *Balantidium entozoon* and *Buxtonella sulcata*. A. *P. coli* from Malmstein (1857); B. *B. entozoon* from Claparéde & Lachmann (1858); and C. *B. sulcata* from Jameson (1926).

**Figure 4:**
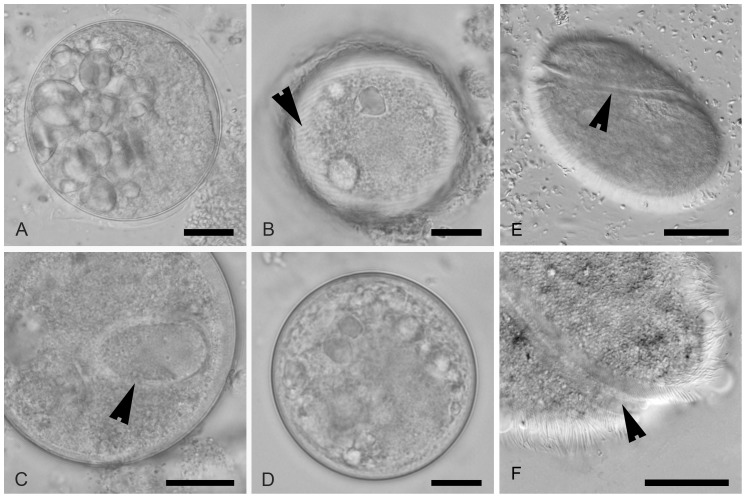
A–D: Comparison of cysts of *Neobalantidium coli*, *Buxtonella sulcata* and a *Buxtonella*-like ciliate; scale bars = 10 µm. A. Cyst of *N. coli* from a domestic pig with visible ingested starch grains inside. B, D. Cysts of *Buxtonella*-like ciliate from an agile mangabey showing the trophozoite with visible rows of cilia (B, arrowhead). C. Cyst of *B. sulcata* from cattle with visible macronucleus (arrowhead). E. Trophozoite of *Buxtonella sulcata* with typical sulcus (arrowhead); scale bar = 20 µm. F. Detail of sulcus of *Buxtonella sulcata* (arrowhead); scale bar = 5 µm.

**Diagnosis:** vestibuliferid ciliate; trophozoite elongated or ovoid (Fig. 3a), rounded posteriorly and narrowing anteriorly, 30–300 µm long and 30–100 µm wide; surface covered with somatic cilia arranged in transverse field; interkinetal ridges present between longitudinal rows of cilia; vestibulum located apically and surrounded by closely packed, which are longer than the somatic ones [15], [26]. Under the pellicle, there are mucocysts that are involved in the synthesis of the paracrystalline and fibrous material utilized in cyst formation [26], [38]. The cyst is spherical or ovoid, 40–60 µm in diameter (Fig. 4a) with a thick and hyaline wall. The trophozoite is present inside the cyst with visible rows of cilia.

**Differential diagnosis:** among the vestibuliferid intestinal ciliates of vertebrates, *Neobalantidium* gen. nov. can be distinguished from most other ciliate genera (including *Balantidium* in poikilothermic vertebrates) by the presence of the cyst stage in the life cycle (Fig. 3a) [15]. The ability to form cysts corresponds with the presence of putative mucocysts. *Buxtonella* spp. also form the cysts that are indistinguishable from those of *Neobalantidium* (Fig. 4 a, c); however, the trophozoites of *Buxtonella* can be distinguished based on the vestibular groove running in a slight curve from the anterior to the posterior end of the body (Fig. 3c) [39]. The vestibular groove being at least as long as one-half of body length is a common feature distinguishing all the other genera within the family Pycnotrichidae [40].

## Discussion

Balantidiasis is classified as a neglected disease [41] and a more or less identical chapter on balantidiasis is included in almost every parasitology textbook. However, many aspects of the infections caused by these ciliates in mammals remain unknown. Although a frequently diagnosed parasitosis of humans until recently [3], [42], balantidiasis is presently rare in developed countries. Most of the reported cases have been attributed to contact with domestic pigs [e.g. 2], but the epidemiology of *B. coli* infections in humans remains controversial. Balantidiasis has also been reported repeatedly in humans without any obvious contact with suids, such as patients from Islamic countries [43]. In contrast, people living in endemic areas with a high prevalence of infections in pigs are often negative or asymptomatic [e.g. 4], [5].

Recently, a high prevalence of *B. coli* has also been recorded in captive African great apes, in contrast to wild populations in which this ciliate is almost absent [7], [44]. Increased contact with reservoir hosts and the diet composition of captive apes were proposed to be the reasons for this discrepancy [7]. As in the situation for humans, the epidemiology of *B. coli* in non-human primates remains unclear.

In the present study, we analyzed our isolates together with reference sequences of *B. coli*-like ciliates from humans, non-human primates and domestic pigs using two phylogenetic markers: SSrRNA and ITS1-5.8SrRNA-ITS2. The most suitable SSrRNA tree having a topology most preferred by the AU test has two major clades, I and II. The latter included trichostomatid ciliates from five families within the orders Vestibuliferida and Entodiniomorphida, corroborating results in which vestibuliferid ciliates assigned as a paraphyletic group with *B. coli*, placed outside this clade [24], [45]. Our molecular phylogenies indicate a situation different from morphology-based taxonomy [39], showing the polyphyletic character of vestibuliferids, into which a number of entodiniomorphid ciliates is inserted. Furthermore, the tree generated from ITS1-5.8SrRNA-ITS2 sequences confirmed this polyphyletic trend at the genus level. Surprisingly, ciliates from mammals traditionally referred to as *B. coli* branched separately in our SSrRNA tree from *B. entozoon* - the nominotypic species of the genus *Balantidium*. The apparent polyphyly of the genus led us to erect a new genus *Neobalantidium*, which we use hereafter.

In the ITS1-5.8SrRNA-ITS2 tree, *B. entozoon* sequences branch as a sister group to *Buxtonella sulcata*. However, this poorly supported branching pattern (52% in the node for sub-clades *B. sulcata* and *B. entozoon* and 61% in the higher node shared by the previously mentioned and cyst-forming ciliates of primates other than apes) might be an artifact due to a low number of ITS sequences from trichostomatid ciliates. This is apparent when ITS and SSU tree topologies are compared. The genetic divergence of *N. coli* and *B. entozoon* is further supported by the formation of cysts in the former and their absence in the latter (and also in other *Balantidium* spp. from amphibians) [27], [46]. In contrast, cyst formation is typical for *B. sulcata* [47] and unidentified ciliates from other primates, such as those described in this study, that both group together with *N. coli* isolates (Fig. 2 b–d).

The phylogenetic analyses presented here represent the first attempt to clarify the position of *B. sulcata*. In the SSrRNA ML tree, *B. sulcata* formed a separate clade together with other yet unidentified ciliates originating from wild agile mangabeys and captive primates (other than apes), which were originally classified as *Balantidium* spp. Throughout the text, we refer to these cyst-forming ciliates as *Buxtonella*-like. However, morphological analyses of their trophozoites are necessary for proper taxonomic evaluation and confirmation of their classification within the genus *Buxtonella* or as a novel genus. Due to ethical considerations, invasive sampling of wild and Zoo primates is complicated, which is why we have been unable to obtain intestinal content.

To date, all cyst-forming ciliates diagnosed in both wild and captive non-human primates, including African great apes, were referred to as *Balantidium coli* [e.g. 6], [7], [48]. However, our data suggest that while captive apes are infected by *N. coli* via unknown means, non-human primates other than apes host *Buxtonella*-like ciliates. Such a scenario could explain the absence of cyst-forming ciliates in wild ranging populations of African great apes and their common presence in other syntopic primates [7], [8], [49]. The presence of *Buxtonella*-like ciliates in primates allows for hypothesizing the occurrence of these pathogens in human populations. The morphology of *Neobalantidium* and *Buxtonella* cysts is almost identical (see Fig. 2) [2], [47], making them indistinguishable from each other in coproscopic diagnostics.

We cannot exclude the possibility of co-infection with both types of cyst-forming ciliates. We most likely detected a mixed infection in an olive baboon from the Sweetwaters Chimpanzee Sanctuary in Kenya. The SSrDNA sequence obtained from this isolate was placed into the *N. coli* clade (JQ073305, see Fig. 1), whereas the ITS sequence branched within the clade of *Buxtonella*-like ciliates (JQ073343, see Fig. 2). *N. coli* was likewise detected in captive chimpanzees in the same sanctuary (SSrDNA: JQ073314, JQ073331-32; ITS regions: JQ073352, JQ073376-77; see Fig. 1, 2) and in domestic pigs from nearby localities (SSrDNA: JQ073333; ITS regions: JQ073378).

Until recently, ITS1-5.8S rRNA-ITS2 had appeared as a favorable marker for research of genetic diversity of trichostomatid ciliates [21], [50], [51]. However, by cloning the products obtained from PCR amplicons showing the multiple sequence signals in chromatograms from a single ciliate cell, Ponce-Gordo et al. [28] has suggested the co-existence of more genetic variants of micronuclear rRNA genes within the same cell of *N. coli*. Based on these facts, they established a novel terminology replacing the term “genotype” by “genetic variant A and B”. Our phylogenies inferred from the sequences of ITS1-5.8S rRNA-ITS2 are concordant with the previous study, as we detected main types A and B and four out of five “types of genetic variants” in our isolates. The presence of both main types was revealed in most of the populations of domestic pigs, whereas only main type A was detected in great apes. Obviously, results of PCR amplification can be biased by the existence of numbers of copies of main variants of ITS1-5.8S rRNA-ITS2 sequences. Similar to previous authors [21], [28], we revealed multiple signals in several chromatographs of *N. coli* sequences and also in *Buxtonella*-like ciliates. Taken together, the present data indicate that ambiguous genetic polymorphisms in the ITS regions of vestibuliferid cyst-forming ciliates are of no taxonomical importance and cannot be used to trace the epidemiology of the infection.

Interestingly, the level of ITS polymorphism differs among the trichostomatid ciliates. The ciliates *Isotricha protostoma* and *Troglodytella abrassarti*, distantly related to *N. coli*, exhibit rather low ITS polymorphism and high host specificity [50], [51]. Transmission of these ciliates is limited, due to the fragile nature of their trophozoites, to close contact between hosts [52], [53]. Higher genetic polymorphism of ITS genes of *N. coli* can result from transmissions facilitated by the resilient cysts of *Neobalantidium*.

Phylogenetically speaking, it is obvious that internal transcribed spacers are not a suitable tool for the study of genetic variability in the vestibuliferid cyst-forming ciliates. Unfortunately, no other hypervariable genes have been exploited in ciliates so far. The broader usage of other genes such as the large ribosomal subunit (LSrRNA), α-tubulin, phosphoglycerate kinase (PGK) and histone genes is unfortunately complicated by several factors: (i) they are highly conserved (histones) [54]; (ii) they provide a very congruent pattern of diversification at the genus level with SSrRNA (LSrRNA) [55]; (iii) sequence data are extremely sparse in GenBank (PGK) [56]; and/or, (iv) there exist multiple paralogs (α-tubulin) [57]. Thus, the necessity of searching for other markers of intraspecific polymorphism of studied cyst-forming ciliates arises, which is important for explaining host specificity and epidemiological aspects.

The proper determination of intestinal ciliates in mammals requires examination of morphological characteristics combined with molecular-phylogenetic data. Obviously, humans are not natural hosts for any intestinal ciliates, and the pathogens are maintained in populations of mammalian reservoir hosts. Our results demonstrate how misleading the coproscopic cyst morphology-based diagnostics of *Neobalantidium* and *Buxtonella* can be, which could also explain the prevailing ambiguity in the epidemiology of these infections (like the occurrence of balantidiasis in areas without pig rearing). So far, no molecular approaches have been applied, either in the diagnostics of balantidiasis in man, or in the broader determination of ciliates detected in clinical cases in different parts of the world. The discovery of novel *Buxtonella*-like ciliates in primates calls for broad molecular-based research addressing the diversity of the agents of balantidiasis in man, not only to describe the source of the infections, but also to set up proper control strategies. In this complex situation, the “old-fashion taxonomy” also matters.

## Supporting Information

Dataset S1 Bayesian phylogenetic tree (PhyloBayes, CAT model) based on SSrDNA sequences. The numbers above branches indicate Bayesian posterior probabilities (CAT model). New sequences are marked with a star.
(PDF)

Dataset S2 Bayesian phylogenetic tree (PhyloBayes, GTR model) based on SSrDNA sequences. The numbers above branches indicate Bayesian posterior probabilities (CAT model)/Bayesian posterior probabilities (GTR model)/PhyML bootstrap computed from 1000 replicates. New sequences are marked with a star.
(PDF)

Dataset S3 Maximum likelihood phylogenetic tree (PhyML, GTR model) based on SSrDNA sequences. The numbers above branches indicate PhyML bootstrap computed from 1000 replicates. New sequences are marked with a star.
(PDF)

Text S1 Overview of research permits and collaborating authorities allowing work at localities in the wild mentioned in the Table 1–3.
(DOC)
