# Supplementary figures and images for: Novel Insights into the Genetic Diversity of Balantidium and Balantidium-like Cyst-forming Ciliates
Source: PLoS Negl Trop Dis. 2013 Mar 28;7(3):e2140. doi: 10.1371/journal.pntd.0002140 (PMC3610628; doi:10.1371/journal.pntd.0002140)

# PhyloBayes CAT

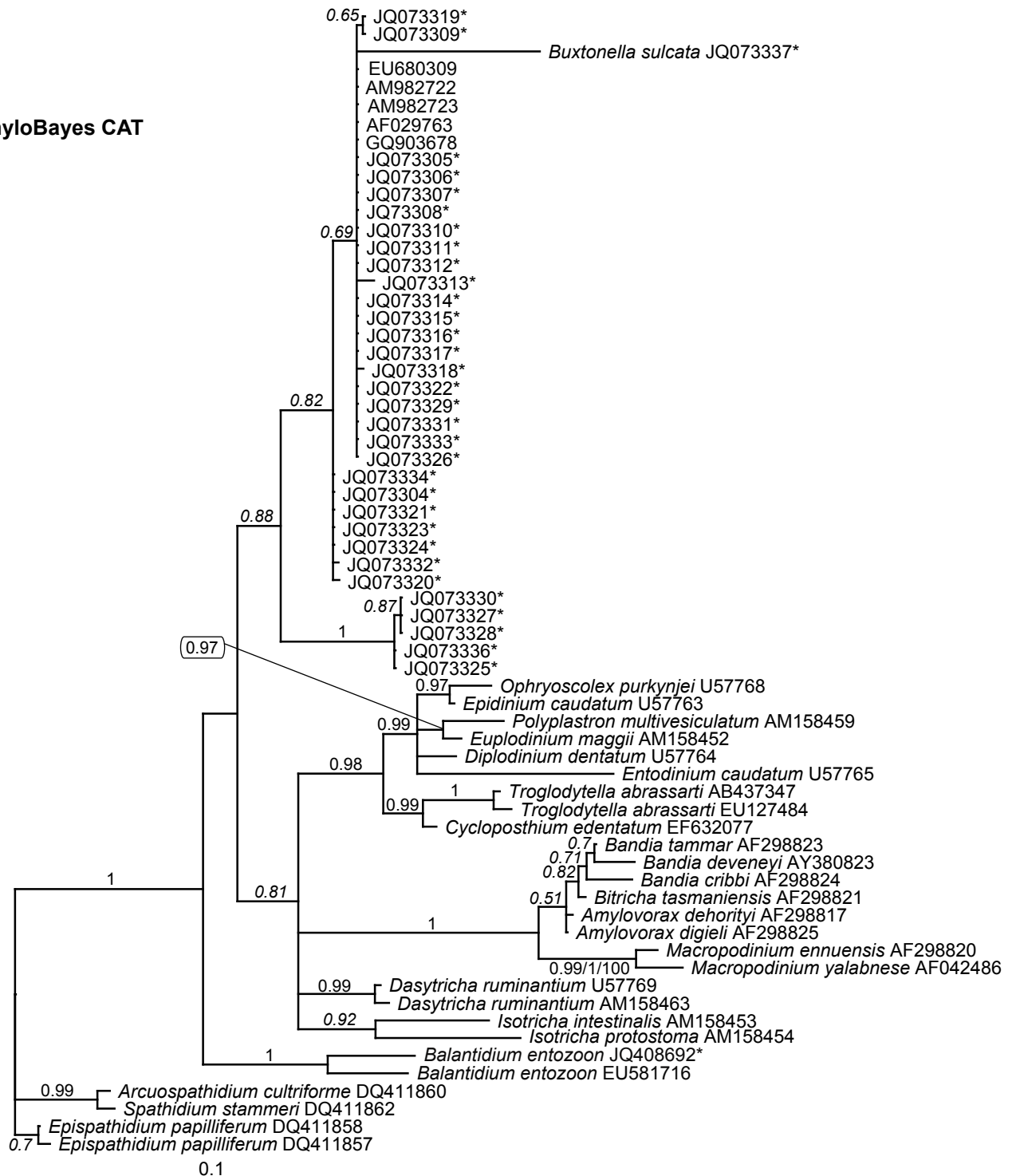

Supplement: Dataset S1 — Bayesian phylogenetic tree (PhyloBayes, CAT model) based on SSrDNA sequences. The numbers above branches indicate Bayesian posterior probabilities (CAT model). New sequences are marked with a star. (PDF) [file pntd.0002140.s001.pdf]

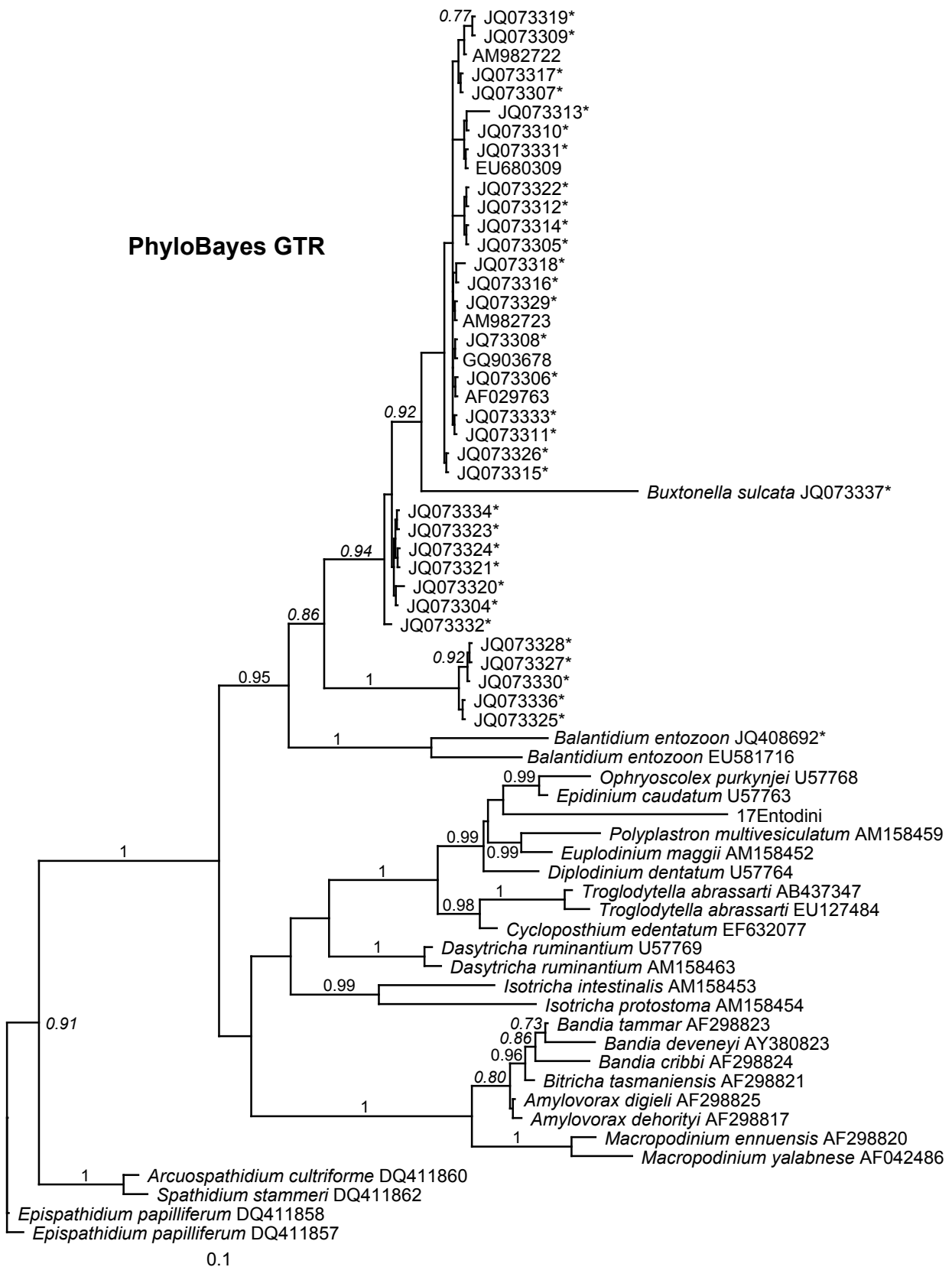

Supplement: Dataset S2 — Bayesian phylogenetic tree (PhyloBayes, GTR model) based on SSrDNA sequences. The numbers above branches indicate Bayesian posterior probabilities (CAT model)/Bayesian posterior probabilities (GTR model)/PhyML bootstrap computed from 1000 replicates. New sequences are marked with a star. (PDF) [file pntd.0002140.s002.pdf]

PhyML GTR

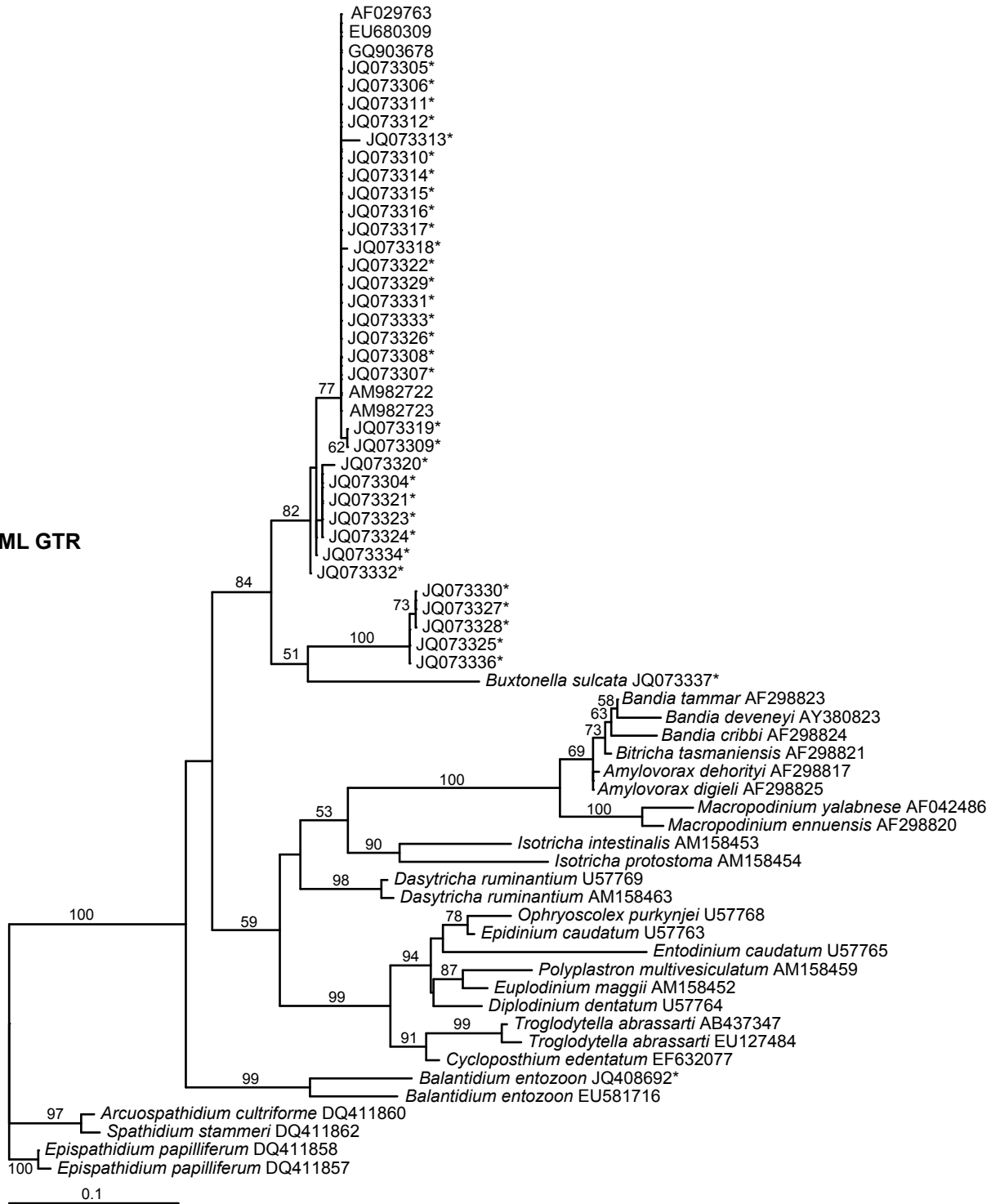

Supplement: Dataset S3 — Maximum likelihood phylogenetic tree (PhyML, GTR model) based on SSrDNA sequences. The numbers above branches indicate PhyML bootstrap computed from 1000 replicates. New sequences are marked with a star. (PDF) [file pntd.0002140.s003.pdf]
