# Supplementary material for: Novel Insights into the Genetic Diversity of Balantidium and Balantidium-like Cyst-forming Ciliates
Source: PLoS Negl Trop Dis. 2013 Mar 28;7(3):e2140. doi: 10.1371/journal.pntd.0002140 (PMC3610628; doi:10.1371/journal.pntd.0002140)
Supplement: Text S1 — Overview of research permits and collaborating authorities allowing work at localities in the wild mentioned in the Table 1–3. (DOC) [file pntd.0002140.s004.doc]

**Research approvals and collaborating authorities**

Our research was approved by several authorities in different countries: the research permits in Central African Republic were obtained from Ministre de l’Education Nationale, de l’Alphabetisation, de l’Enseignement, Superieur et de la Recherche and our work adhered to the research protocol defined by Dzanga-Sangha Protected Areas and granted by the Government of the Central African Republic and the World Wildlife Fund; in Cameroon, research was conducted by Pandrillus foundation and followed the protocol of Limbe Wildlife Centre; in Kenya, research was approved by ethical committee of Ol Pejeta Conservancy and Kenyan Wildlife Service; in Republic of Congo, the permits were granted by the Ministre de Développement Durable, de l'Economie Forestier et de l'Environnement (MDDEFE) in charge of the Protected Areas in the Republic of Congo for research at Conkouati-Douli National Park and we would like to thank the MDDEFE conservator and the Conseiller Technique Principale of the Wildlife Conservation Society (WCS) for their support on site; in Madagascar, the sampling of domestic pigs was conducted in collaboration with responsible officers of the Service for the Combatting of Animal Illness; in Romania, the permits (12309/ARBDD/4.7.2011) for collections of samples of edible frogs were granted by the Administratia Rezervatiei Biosferei Delta Dunarii.
